# Viral dosing of influenza A infection reveals involvement of RIPK3 and FADD, but not MLKL

**DOI:** 10.1038/s41419-021-03746-0

**Published:** 2021-05-11

**Authors:** Teodora Oltean, Emily Van San, Tatyana Divert, Tom Vanden Berghe, Xavier Saelens, Jonathan Maelfait, Nozomi Takahashi, Peter Vandenabeele

**Affiliations:** 1 VIB-UGent Center for Inflammation Research, Ghent, Belgium; 2 Department of Biomedical Molecular Biology, Ghent University, Ghent, Belgium; 3 Department of Biomedical Sciences, University of Antwerp, Antwerp, Belgium; 4 VIB-UGent Center for Medical Biotechnology, Ghent, Belgium; 5 Department of Biochemistry and Microbiology, Ghent University, Ghent, Belgium

**Keywords:** Cell death, Cell death and immune response, Infection

## Abstract

RIPK3 was reported to play an important role in the protection against influenza A virus (IAV) in vivo. Here we show that the requirement of RIPK3 for protection against IAV infection in vivo is only apparent within a limited dose range of IAV challenge. We found that this protective outcome is independent from RIPK3 kinase activity and from MLKL. This shows that platform function of RIPK3 rather than its kinase activity is required for protection, suggesting that a RIPK3 function independent of necroptosis is implicated. In line with this finding, we show that FADD-dependent apoptosis has a crucial additional effect in protection against IAV infection. Altogether, we show that RIPK3 contributes to protection against IAV in a narrow challenge dose range by a mechanism that is independent of its kinase activity and its capacity to induce necroptosis.

## Introduction

Influenza A viruses (IAVs) are negative-stranded RNA viruses that belong to the *Orthomyxoviridae* family. IAVs can cause influenza in birds and some mammals, including humans. In mammals, IAV typically infects lung epithelial cells and replicates in the nucleus where the virus also hijacks the host’s transcription machinery^1^. Programmed cell death (PCD) plays a role as a cell-autonomous defense mechanism by which infected cells die resulting in limited viral replication and spreading^2^. Apoptosis is the major type of PCD upon in vitro infection of cells with IAV^3,4^, but necroptosis and pyroptosis were also shown to be elicited^5–9^. Many viruses have evolved genes that can block apoptosis such as viral inhibitor of caspase-8 activation (vICA) and viral mitochondria-localized inhibitor of apoptosis (vMIA) in mouse cytomegalovirus (MCMV)^10^. These viral proteins allow viruses to escape PCD. From an evolutionary point of view necroptosis is an alternative cell death mechanism that promotes the killing of host cells that acquired resistance to caspase-8-mediated cell-extrinsic apoptosis and can also restrict pathogen replication^11^. Receptor-interacting serine/threonine-protein kinase 3 (RIPK3) is activated by RIP Homology Interaction Motif (RHIM)-dependent recruitment to RIPK1, TIR-domain-containing adapter-inducing interferon-β (TRIF) or Z-nucleic acid Binding Protein-1 (ZBP1)^10,12^. This results in RIPK3 autophosphorylation and phosphorylation of its substrate mixed lineage kinase domain-like pseudokinase (MLKL)^13,14^ which destabilizes the plasma membrane and executes necroptosis^15,16^.

RIPK3 was reported to be crucial for the protection against IAV. Some studies report that *Ripk3*^−/−^ mice are more susceptible to IAV infection suggesting the importance of this protein in the in vivo protection against IAV infection^5,7,17^. During IAV replication virally-derived RNA molecules are sensed by ZBP1, which binds to RIPK3 to initiate parallel cell modality outcomes of pyroptosis, apoptosis, and necroptosis within the population of infected cells, collectively coined PANoptosis^18^. Intracellular viral replication also activates RIPK3 to drive PCD^19^. In vitro, RIPK3 was shown to activate in parallel both MLKL-mediated necroptosis and Fas-associated protein with death domain (FADD)-induced apoptosis in IAV infected cells^7^. It was also shown that replicating IAV activates the RNA sensor ZBP1 in the nucleus, followed by RIPK3 activation and nuclear MLKL phosphorylation resulting in nuclear envelope disruption, and necroptosis^20^. However, the involvement of necroptosis in IAV infection remains controversial in vivo. While some studies report that RIPK3-mediated necroptosis may negatively impact on survival by inducing immunopathology^21^, others show that necroptosis does not affect survival and that it functions as a protective backup mechanism only when the proteolytic inactivity of caspase-8 is compromised^16,20^. We hypothesized that these apparent discrepancies in the susceptibility in terms of survival between of *Ripk3*^−/−^ mice to IAV infection may be due to differences in the viral dose used to challenge the mice. Moreover, previous conclusions related to the role of necroptosis in IAV infection through the use of *Ripk3*^−/−^ mice should be taken with caution as RIPK3 is required to induce both apoptosis and necroptosis downstream of ZBP1^6^. While some studies suggest that necroptosis ensures protection against IAV in murine^12,21^ others found that necroptosis is only functioning as a backup mechanism when apoptosis is blocked^5^. Furthermore, in response to an IAV challenge dose that was lethal for wild-type (WT) mice, a significant fraction of the *Mlkl*^*/−*^ mice survived this challenge dose^20^. These discrepancies between studies that report increased susceptibility of RIPK3-deficient mice to IAV infection^7,17,20^ and those that described no change in susceptibility between *Ripk3*^−/−^ mice and WT controls^21^, may be due to differences in the viral dose used to challenge the mice. Here, we investigated the susceptibility of RIPK3-deficient mice to a range of viral doses and examined the possible involvement of either RIPK3-induced necroptosis or apoptosis. Our results confirm that RIPK3 is implicated in a protective response during viral infection but only at a limited range of IAV challenge doses. Moreover, we demonstrate that the protective effect is independent of RIPK3 kinase activity and its downstream substrate MLKL. We additionally show that FADD is crucial for protection against limited IAV challenge doses. We therefore propose that RIPK3 platform activity-dependent and FADD-mediated apoptosis partially protect laboratory mice against IAV-infection in vivo.

## Results

### RIPK3-mediated protection is only observed at medium dose of IAV infection in vivo

**Fig. 1:**
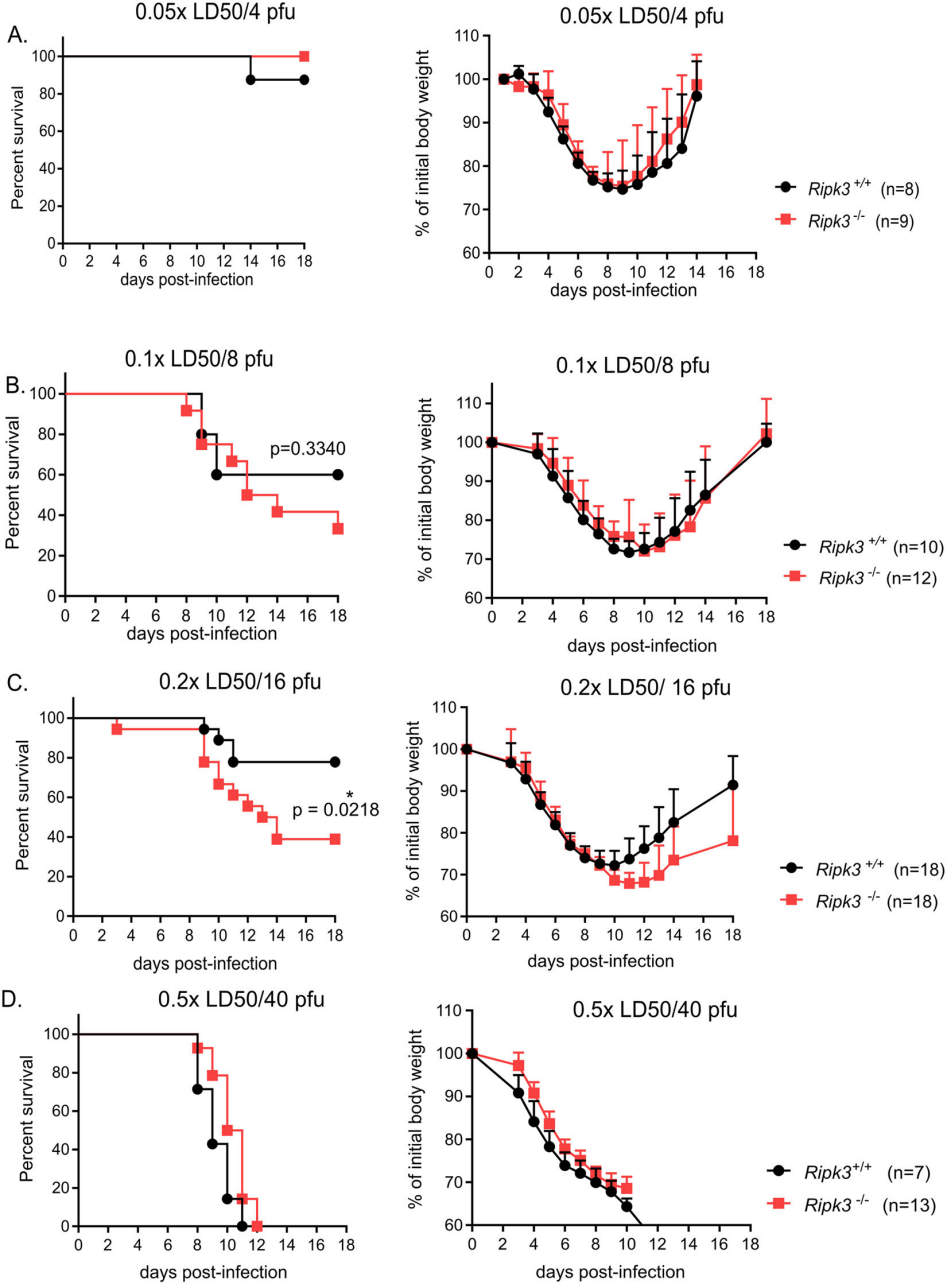
RIPK3 is partially required for protection at medium IAV dose in vivo. Survival analysis and body weight loss of age-matched *Ripk3*^−/−^ and *Ripk3*^*+/+*^ mice infected intranasally with IAV is shown at very low dose: 0.05x LD_50_/4 pfu; (**a**), low dose: 0.1x LD_50_/8 pfu *p* value = 0.3340 (**b**), medium dose: 0.2x LD_50_/16 pfu p value= 0.0218 (**c**) and high dose: 0.5x LD_50_/40 pfu (**d**). Data were pooled from 2 (panel A and B) or 3 (**c**, **d**) independent experiments. Bodyweight curves are shown as mean ± SD. Survival curves were plotted for indicated groups and evaluated statistically according to Kaplan–Meier. A log-rank test verified significant differences between *Ripk3*^*+/+*^ and *Ripk3*^−/−^ mice (GraphPad Prism 7). *p < 0.05.

To assess the protective role of RIPK3 against IAV challenge in vivo, we challenged WT littermates and RIPK3-deficient mice with different doses of PR8 virus, a commonly used human-origin laboratory strain of IAV. We exposed the mice to different infection doses: very low IAV dose (0.05x LD_50_ or 4 pfu), low dose (0.1x LD_50_ or 8 pfu), medium IAV dose (0.2x LD_50_ or 16 pfu) and high IAV dose (0.5x LD_50_ or 40 pfu). Following a very low challenge dose (0.05x LD_50_/4 pfu), *Ripk3*^−/−^ and WT mice displayed similar mortality and most of the mice survived the challenge (as defined in Materials and Methods) (Fig. 1a, b). Doubling the challenge dose from 0.05x LD_50_/4 pfu to 0.1x LD_50_/8 pfu revealed a tendency towards increased sensitivity of RIPK3-deficient mice, though not statistically significant. For the medium viral dose of infection (0.2x LD_50_/16 pfu) the susceptibility of *Ripk3*^−/−^ mice is significantly potentiated (p value= 0.0218) compared to *Ripk3*^*+/+*^ littermates. Infection with high viral dose (0.5x LD_50_/40 pfu) leads to 100% lethality in both *Ripk3*^−/−^ mice and *Ripk3*^*+/+*^ littermates (Fig. 1d). Altogether, these results suggest that, in contrast to many studies that report only one dose of viral infection, the involvement of RIPK3 for protection against IAV infection is partial and can only be demonstrated within a narrow dose-range, which is in our case the medium dose of 0.2x LD_50_/16 pfu. For this dose, the surviving *Ripk3*^−/−^ mice seem to suffer slightly more weight loss compared to WT controls. This is most probably due to slower recovery of the RIPK3-deficient mice and not due to enhanced morbidity during the first 9–10 days of infection. We do not observe any shift in the bodyweight loss curve for the other infection doses suggesting that RIPK3 only affects the mortality threshold (i.e. the ethical endpoint) of the mice (Fig. 1).

### MLKL does not protect against different IAV challenge doses in vivo

**Fig. 2:**
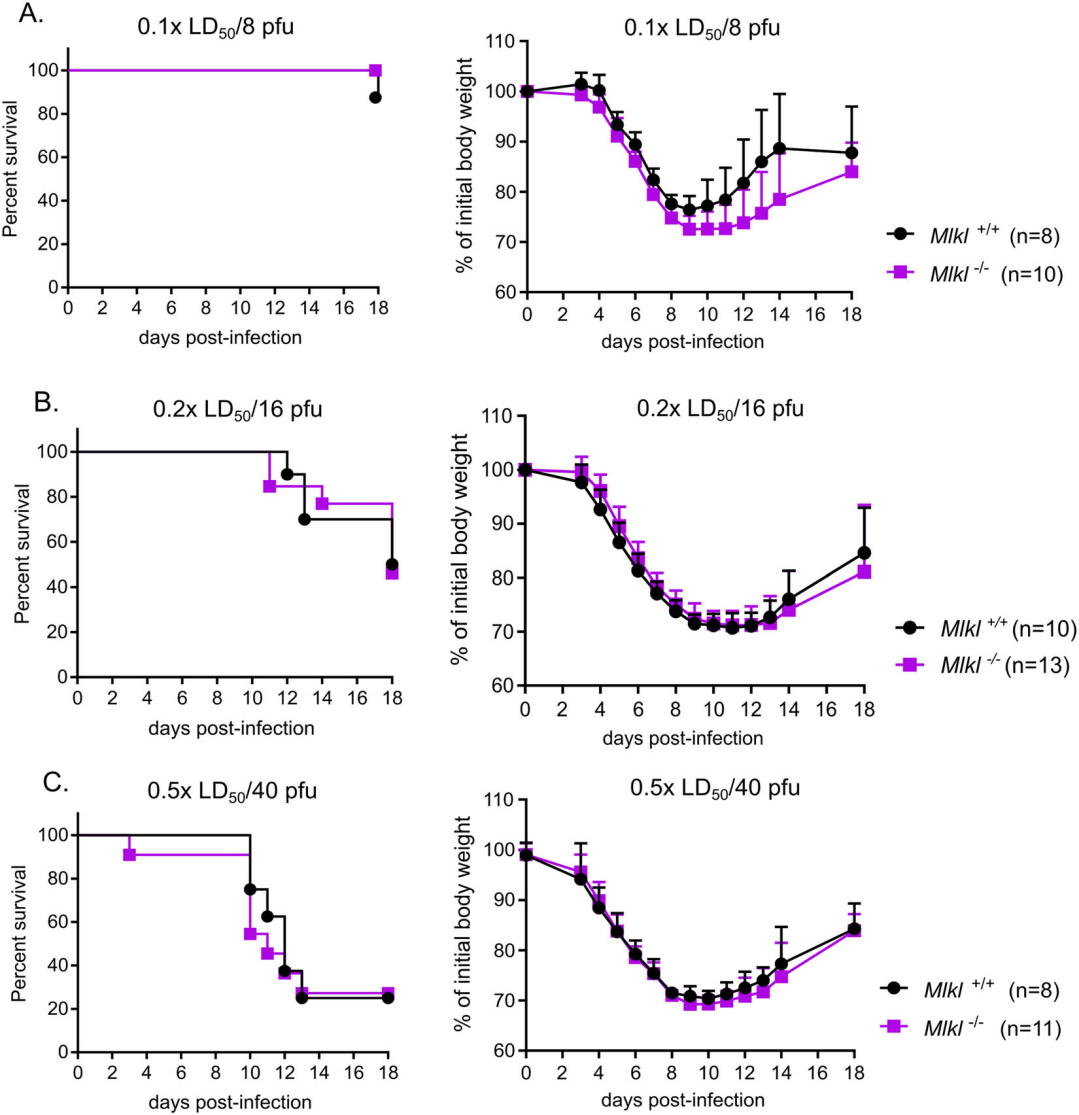
MLKL is not required for partial protection against IAV infection at different viral doses. Survival analysis and bodyweight loss of age-matched *Mlkl*^−/−^ and *Mlkl*^*+/+*^ infected intranasally with IAV is shown at each infection: low dose: 0.1x LD_50_/8 pfu (**a**), medium dose: 0.2x LD_50_/16 pfu (**b**) and high dose: 0.5x LD_50_/40 pfu (**c**). Data were pooled from 2 (**a**, **c**) or 3 (**b**) independent experiments. Bodyweight curves are shown as mean ± SD. Survival curves were plotted for indicated groups and evaluated statistically according to Kaplan–Meier (GraphPad Prism 7). NS not significant.

In view of the reported protective role of the RIPK3-MLKL axis against IAV infection of cells in vitro^7^ and because of the protective role of RIPK3 in vivo at certain infection doses of IAV (Fig. 1), we wanted to clarify whether RIPK3 acted through activation of MLKL during in vivo IAV infection. Therefore, we challenged MLKL-deficient mice and their littermate controls with low (0.1x LD_50_/ 8 pfu) medium (0.2x LD_50_/16 pfu) and high IAV doses (0.5x LD_50_/40 pfu). Survival analysis and bodyweight loss curves are shown in Fig. 2a–c. *Mlkl*^*+/+*^ and MLKL-deficient mice displayed the same bodyweight loss and survival at any of the challenge doses used, excluding a role for MLKL downstream of RIPK3 during protection against medium-dose IAV infection. This confirms previously published results where deficiency of MLKL alone did not affect the lethal sensitivity during IAV infection^5,16^.

### RIPK3 platform function but not its kinase activity is required for protection against medium IAV infection dose in vivo

**Fig. 3:**
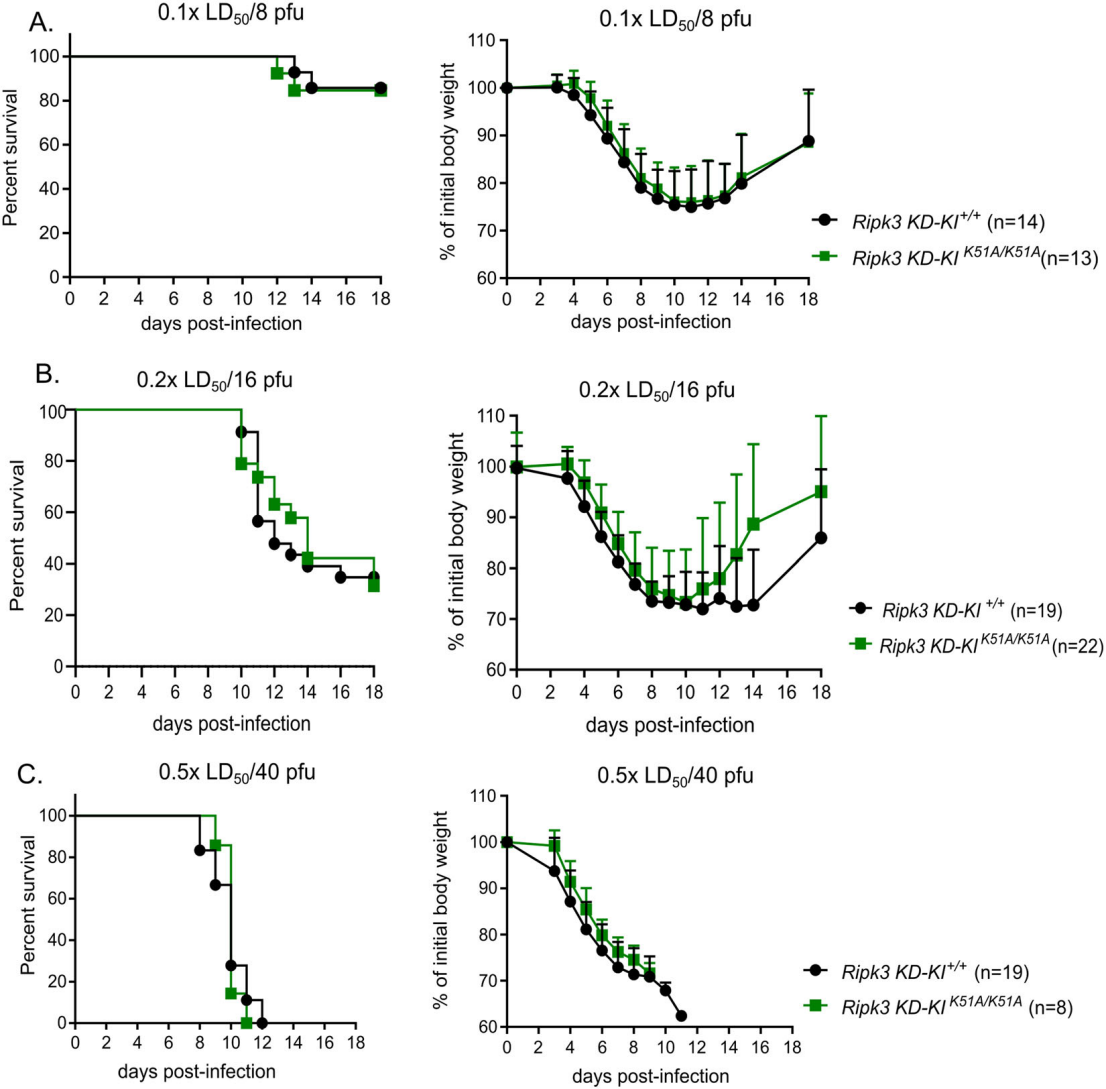
RIPK3 kinase activity is not required for partial protection against IAV infection at different viral doses. Survival analysis and bodyweight loss of age-matched *Ripk3 KD-KI*^*K51A/K51A*^ and *Ripk3 KD-KI*^*+/+*^ mice infected intranasally with IAV is shown at each infection dose: low dose: 0.1x LD_50_/8 pfu, (**a**), medium dose: 0.2x LD_50_/16 pfu, (**b**) and high dose: 0.5x LD_50_/40 pfu (**c**). Data were pooled from 2 (panel A), 3 (**c**) or 5 (**b**) independent experiments. Bodyweight curves are shown as mean ± SD. Survival curves were plotted for indicated groups and evaluated statistically according to Kaplan–Meier (GraphPad Prism 7). NS not significant.

To examine if the kinase activity of RIPK3 is important for the protection against IAV, we infected with different IAV doses (low, medium, high, as defined above) mice that lack the kinase activity of RIPK3, i.e. RIPK3K51A knock in mice. The bodyweight loss and lethality of *Ripk3 KD-KI*^*K51A/K51A*^ mice infected with low (0.1x LD_50_/8 pfu), medium (0.2x LD_50_/16 pfu), and high viral dose (0.5x LD_50_/40 pfu) are not statistically significantly different from their littermates, the *Ripk3 KD-KI*^*+/+*^ (Fig. 3a–c). This suggests that that the kinase activity of RIPK3 apparently is not required for the protective effect during IAV infection. This observation is in line with the absence of a sensitizing phenotype in *Mlkl*^−/−^ mice following IAV infection (Fig. 2a–c). Altogether our findings favor a model in which the platform function of RIPK3 rather than its RIPK3 kinase activity and consecutive MLKL-mediated necroptosis is implicated in the protective effect against the medium dose IAV infection in vivo.

### FADD is required for RIPK3-mediated protection against low and medium IAV doses in vivo

**Fig. 4:**
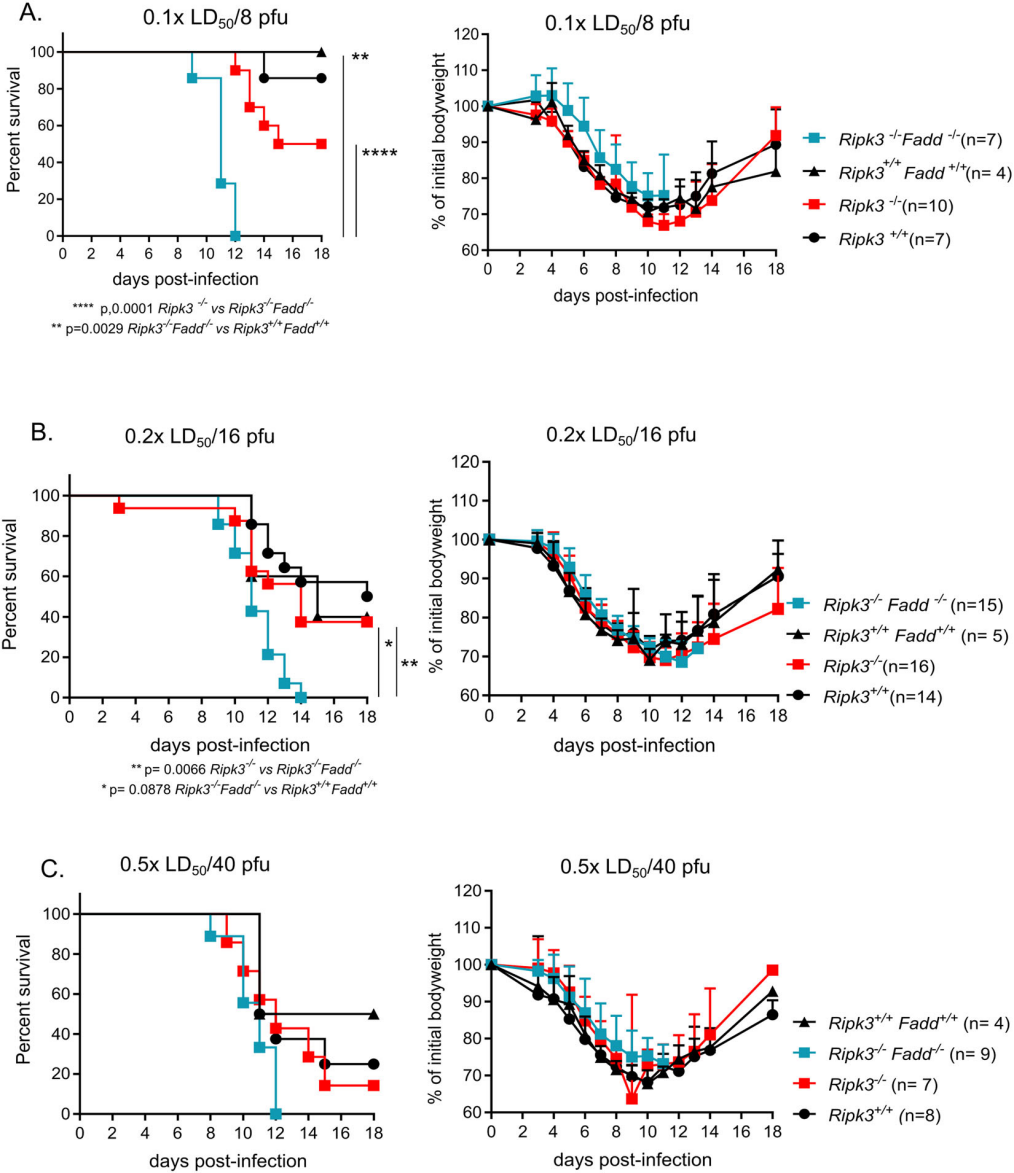
FADD is required against low and medium IAV doses in vivo. Survival analysis and bodyweight loss of age-matched *Ripk3*^−/−^*Fadd*^−/−^ DKO and WT controls *Ripk3*^*+/+*^ *Fadd*^*+/+*^ as well as single *Ripk3*^−/−^ and *Ripk3*^*+/*+^ mice infected intranasally with PR8 are shown: low dose: 0.1x LD_50_/8 pfu (**a**), medium dose: 0.2x LD_50_/16 pfu (**b**) and high dose: 0.5x LD_50_/40 pfu (**c**). Data were pooled from 2 (**a**) or 3 independent experiments (**b**, **c**). Bodyweight curves are shown as mean ± SD. Survival curves were plotted for indicated groups and evaluated statistically according to Kaplan–Meier (GraphPad Prism 7). NS not significant; *p < 0.05, **p < 0.01, and ***p < 0.001.

RIPK3 can function as a platform that stimulates apoptosis^22^. Since *Fadd*^*−/−*^mice die *in utero*^23^ due to excessive RIPK3/MLKL-mediated necroptosis in multiple tissues^24^, we infected *Ripk3*^−/−^*Fadd*^−/−^ double knock out (DKO) mice with low (0.1x LD_50_/8 pfu), medium (0.2x LD_50_/16 pfu), and high viral dose (0.5x LD_50_/40 pfu) of IAV infection to study the possible contribution of the FADD-caspase-8 apoptotic axis. These DKO mice are born at normal Mendelian frequency and develop normally^25^. Their survival and bodyweight loss were monitored up to 18 days post-IAV infection (Fig. 4a–c). Interestingly, already at a low dose of infection (0.1x LD_50_/8 pfu) *Ripk3*^−/−^*Fadd*^−/−^ DKO mice showed enhanced susceptibility to IAV compared to their littermates (*p* value= 0.0878) and compared to RIPK3-deficient mice (*p* value =0.0066). All *Ripk3*^−/−^*Fadd*^−/−^ DKO mice died after the challenge, none of them being able to recover from infection-induced morbidity compared to the control mice (Fig. 4a–c). These results resemble the previously reported phenotype of sensitization following IAV infection of *Fadd*^−/−^*Mlkl*^−/−^ DKO^7^ or the *Casp8*^*DA*^*Mlkl*^−/−^ mice^5^. This suggests the importance of FADD-dependent apoptosis in the protection against IAV infection. Moreover, the susceptibility of *Ripk3*^−/−^ *Fadd*^−/−^ double knock-out mice is further increased compared to RIPK3-deficient mice for the same doses, suggesting that residual RIPK3-independent and FADD-dependent apoptosis mediates protection against IAV independent from RIPK3 or MLKL (Fig. 4a).

## Discussion

Several reports have highlighted the role of RIPK3 in the protection against IAV infection^5,7,17,21^. Furthermore, it was shown that genetic deletion of *ripk3* rescued cellular inhibitor of apoptosis 2 (cIAP2)-deficient mice from influenza-induced lethality via RIPK3-mediated necroptosis^21^. In contrast, it has also been reported that infection with a sublethal IAV dose resulted in similar susceptibility of RIPK3-deficient mice as WT mice^21^, suggesting that the in vivo protective role of RIPK3 against IAV challenge infection may be dose dependent. This absence of phenotype of RIPK3-deficient mice is comparable to what we observe when mice are infected with a very low and low IAV dose. This observation is in contrast with other studies that reported that RIPK3-deficient mice were highly susceptible to a sublethal viral (50 pfu) dose as well as to a lethal dose (90 pfu, 1x LD_50_) of IAV, including the PR8 virus strain that we used here^7,17^. Therefore, the opposing conclusions in the literature with regard to the protective role of RIPK3 following IAV challenge might be very likely explained by discrepancies in doses of IAV challenge leading to between reports emphasizing its protective role^7^ or those dismissing a role for RIPK3^17^. Importantly, in our study we reconcile these observations by demonstrating that depending on the challenge dose range, RIPK3 can contribute or not to protection against IAV infection in vivo.

Next, we addressed whether the role of RIPK3 in partial IAV protection could be attributed to RIPK3-mediated MLKL phosphorylation and necroptosis execution. MLKL-deficient mice challenged with different IAV infection doses (low, medium, and high) did not exhibit increased susceptibility compared to WT littermates suggesting that necroptosis execution mechanism apparently is not implicated in IAV protection in vivo, confirming previously published data^7,20^. This result prompted the question whether the RIPK3 kinase activity would be implicated or not in the in vivo protection. To this end, we challenged RIPK3 kinase activity deficient *Ripk3 KD-KI*^*K51A/K51A*^ mice with different IAV doses. These mice responded like control littermates excluding the RIPK3 kinase involvement in the protective process to IAV in murine hosts. We noticed that not all littermate controls respond equally to the viral challenge. For instance, the *Ripk3 KD-KI*^*+/+*^ and the *Mlkl* ^*+/+*^ mice are more susceptible than *Ripk3*^*+/+*^ for the same viral challenge dose (0.2x LD_50_). The use of littermate controls is essential when comparing the outcome of an immunity-related stimulus between genetically modified strains^26,27^. Particularly when evaluating viral susceptibility of KO mice, specific littermate controls should be used in each experiment. The Kaplan-Meier survival curves of all the wild-type control littermates for every knockout line used in this study is shown in Supplementary Fig. 1. When we put all results of wild-type littermates with wild-type alleles in one figure, we noticed significant variation in survival following infection with the high dose (0.5x LD50/40 pfu). This illustrates genetic and experimental differences over the years between control littermates with wild-type alleles, reinforcing the point that conclusions can only be drawn using the appropriate littermates. In all figures, we pooled data from mutant mice with appropriate wild type littermate controls each time performed in the same experimental setup as indicated in the figure legends.

Besides necroptosis, RIPK3 has also been reported to induce NF-kB activation upon overexpression^28^, is involved in cytokine production^29^, can promote activation of apoptosis as a kinase-dead platform^22,30^ and is implicated in NLR family pyrin domain containing 3 (NLRP3) inflammasome activation in response to polyinosinic:polycytidylic acid [poly(I:C)] and lipopolysaccharides^22,31–33^ and RNA viruses^34^. The role in NF-kB activation has been controversial, since experiments based on cells from *Ripk3*^−/−^ revealed that RIPK3 is dispensable for normal NF-κB, signaling by the B-Cell and T-Cell Receptors, tumor necrosis factor receptor 1 (TNFR1), and toll-like receptor (TLR) 2 and TLR4^28^. RIPK3 was shown to activate the NLRP3 inflammasome in IAV infected cells^6^. Depending on the biological context, RIPK3 was shown to be dispensable for inflammasome activation by RNA viruses^35^ or required for the NLRP3 inflammasome induced by LPS in which case the kinase activity is required as it can be inhibited by the RIPK3 kinase inhibitor GSK872^36^. Inflammasome activation is described to be involved in the protection against a wide variety of viruses such as IAV, herpes simplex virus 1 (HSV-1), West Nile virus (WNV), Sendai virus, respiratory syncytial virus (RSV), encephalomyocarditis virus (EMCV). However, two independent studies showed that mice lacking components of the NLRP3 inflammasome pathway, such *as Caspase-1/11* DKO mice and *Nlrp3* KO mice infected with a sublethal IAV dose did not exhibit increased susceptibility compared to their WT littermates suggesting that these pathways apparently are not crucial for protection in this context^21,37^.

**Fig. 5:**
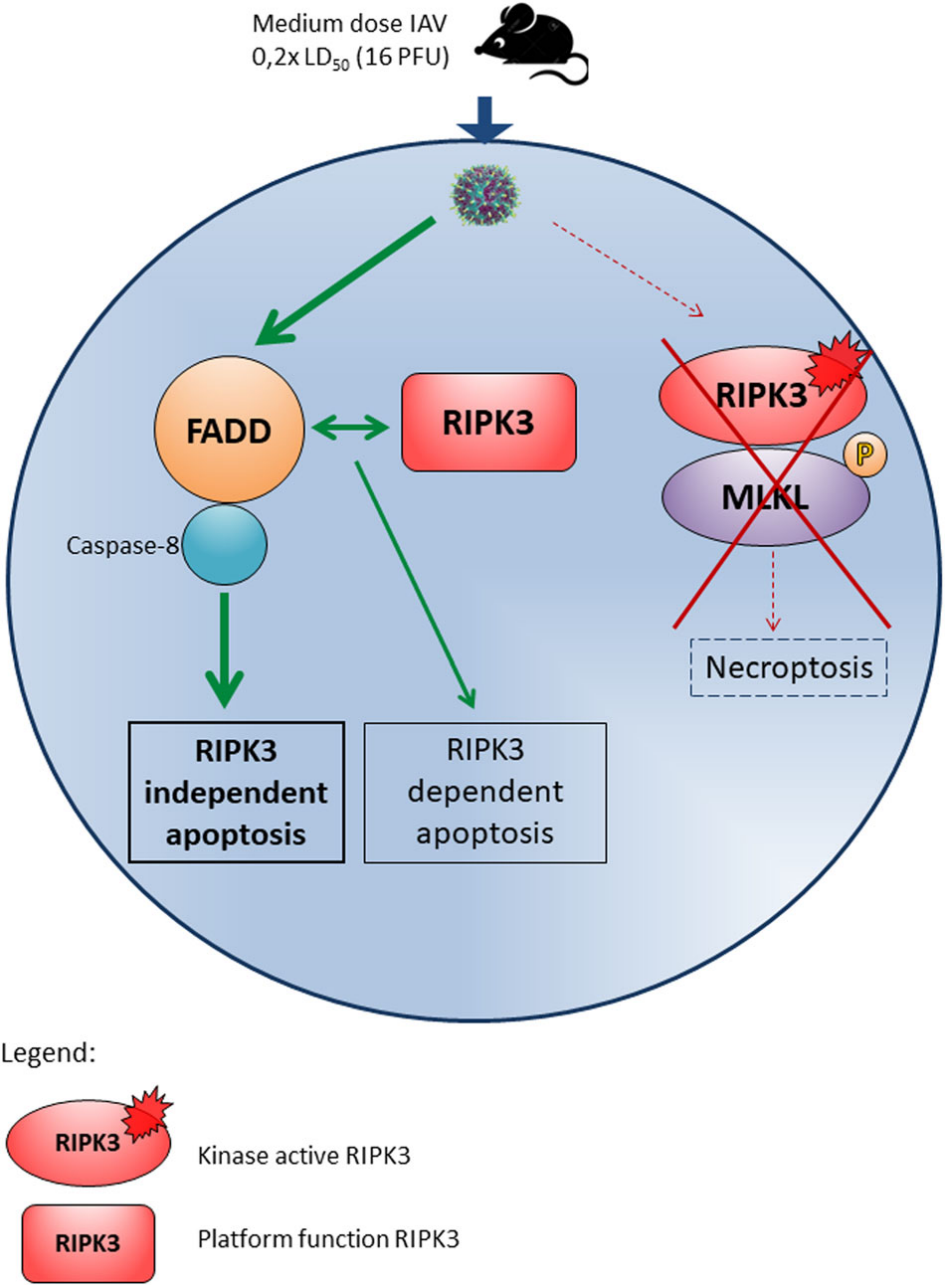
Graphic summary: involvement of FADD and RIPK3 in protection at different doses of IAV challenge. After low to medium IAV challenge dose in vivo, intracellular IAV activates FADD to drive apoptosis of infected cells and protects the host (main pathway involved in protection). RIPK3 is not essential to activate FADD-dependent apoptosis post IAV infection. The platform function of RIPK3 can associate with FADD and caspase-8 to drive apoptosis (to a lesser extent than the main pathway which is RIPK3-independent) or other cell death-independent mechanism for protection. The kinase active RIPK3 and downstream MLKL are not involved in the protection against IAV.

As mentioned above, the platform function of RIPK3 is implicated in the induction of apoptosis through the recruitment of RIPK1, FADD, and caspase-8^22,38^. In order to examine this apoptosis-connected platform function of RIPK3, we challenged *Ripk3*^−/−^ *Fadd*^−/−^ DKO mice to assess susceptibility to IAV infection. Our results reveal that even a low viral infection dose is sufficient to kill all *Ripk3*^−/−^*Fadd*^−/−^ DKO mice shortly after infection even to a higher extend than *Ripk3*^−/−^ mice, showing indicating that FADD-mediated apoptosis is implicated. This strong sensitization of IAV-mediated lethality in the absence of both FADD and RIPK3 (this paper) or both FADD and MLKL^7^, or both caspase 8 protease activity and MLKL^5^ all argue for the crucial involvement of apoptosis rather than necroptosis in controlling the life or death outcome following IAV infection. Moreover, since the absence of both FADD and RIPK3 sensitizes to lethality following IAV infection even more than loss of RIPK3 kinase activity, a paradigm emerges in which beyond RIPK3 platform-mediated apoptosis also other apoptotic pathways are probably implicated with even higher protective potential. Although we do not show any mechanistical connection between FADD and RIPK3, our results show that in addition to the RIPK3-mediated protection, FADD exerts an additional protective effect. Furthermore, it was described that cytotoxic CD8^+^ T cells induce Fas-mediated apoptosis to destroy infected cells of the lung epithelia. This process was shown to be crucial for limiting in vivo viral pneumonia due to IAV infection^39^. Also, caspase-8 is critical for proper TLR and NF-κB involved in immune defense and its loss in B cells is associated with a reduced anti-viral antibody response^40^. Figure 5 summarizes a model on the involvement of FADD and RIPK3 in the protection against limited doses of IAV infection. In IAV infection, apoptosis is the primary host cell defense mechanism inducing viral clearance and limiting viral spread^41^. A previous study showed an increase in virus spread, enhanced pulmonary edema, and alveolar damage in influenza A virus-infected *Ripk3*^−/−^ compared to WT mice. Moreover, the RIPK3-deficient mice also had significantly lower CD3^+^ and IAV-specific CD8^+^ T cells^7^.

In conclusion, we propose a paradigm by which RIPK3 platform-dependent and RIPK3-independent FADD-mediated apoptosis are crucial for protection against IAV in vivo, and that MLKL-mediated necroptosis is dispensable at least when the activity of caspase-8 is not compromised. Moreover, our results emphasize the absolute need to examine sensitivity to different viral doses in vivo in order to obtain a comprehensive view on the role of cell death molecules in viral protection. Indeed, often published statements are made based on the use of one single infection dose in vivo possibly leading to incomplete conclusions that only apply to particular IAV infection doses. Moreover, the use of littermate controls is the best good practice while working with mice containing mutated alleles. Even then, small phenotypic differences can occur between different littermate control mice with wild type alleles due to passenger mutations^27^.

## Material and methods

### Mice

*Ripk3*^*−/−*^ were kindly provided by Dr. Vishva Dixit (Genentech, San Francisco)^38^, *Mlkl*^*−/−*^ by Dr. Alexander Warren and Dr. James Murphy^42^ and *Ripk3 K51A kinase dead knock-in* (*Ripk3 KD-KI*^*K51A/K51A*^) mice by Dr. John Bertin by GlaxoSmithKline^22^. The *Ripk3*^*−/−*^ animals were congenic to the C57BL/6 N background, while all other strains were of the C57BL/6 J background, and were therefore compared with the appropriate littermate controls. *Ripk3*^−/−^ mice were housed in individually ventilated cages in a conventional animal house. The other mice were bred and housed in the SPF facility in individually ventilated cages. Three weeks prior to the experiment all mice were transferred to the conventional animal house and allowed to go through a quarantine and accommodation period of minimum 3 weeks before the infection experiment. Littermate controls of *Ripk3*^−/−^, *Mlkl*^−/−^*, Ripk3*^−/−^*Fadd*^−/−^ and *Ripk3 KD-KI*^*K51A/K51A*^ were used in each experiment. In all experiments, 10–15-week-old mice were used. All animal experiments were done under conditions specified by law (European Directive and Belgian Royal Decree of November 14, 1993) and approved by the Institutional Ethics Committee on Experimental Animals.

### Viral infection

Age-matched mice were anesthetized with a cocktail of 87,5 mg/kg ketamine and 12,5 mg/kg xylazine intraperitoneally and infected intranasally with 50 μl/20 g phosphate-buffered saline (PBS) containing different doses of influenza virus A/PR/8/34^43^, as described in the legends. The plaque-forming units (pfu) were determined by plaque assay on Madin-Darby Canine Kidney (MDCK) cells, as described previously^44^. The LD_50_ of the viral batch was determined on BALB/c mice and 1x LD_50_ represented 80 pfu, as determined in the lab of Prof. Saelens. Although the LD_50_ is not referring to 50% of death in the mice that were used in this study, the nomenclature is used together with the pfu to have a Supplementary information regarding the power of the virus in vivo. This terminology is often used in the papers cited here. Age- and sex-matched mice were challenged with 0.05x LD_50_ (4 pfu), 0.1x LD_50_ (8 pfu), 0.2x LD_50_ (16 pfu) or 0.5x LD_50_ (40 pfu) and monitored for survival and weight loss over a period of at least 18 days. We used the following 4 scores of clinical symptoms: 0 = no visible signs of disease; 1 = slight ruffling of fur; 2 = ruffled fur, reduced mobility; 3 = ruffled fur, reduced mobility, rapid breathing; 4 = ruffled fur, minimal mobility, huddled appearance, rapid and/or labored breathing indicative of pneumonia and body temperature below 32 °C. For the combination of body weight loss by 30% and a clinical score 4 the mice were considered moribund and euthanized by CO_2_ asphyxiation or cervical dislocation (EC2016–17).

### Statistics

All the survival data were analyzed by Kaplan-Meier survival analysis using the software Prism 7.04 (GraphPad), and p‐values were calculated.

## Supplementary information

Supplementary figure 1

Legend for supplementary figure 1
