# Supplementary figures and images for: Viral dosing of influenza A infection reveals involvement of RIPK3 and FADD, but not MLKL
Source: Cell Death Dis. 2021 May 11;12(5):471. doi: 10.1038/s41419-021-03746-0 (PMC8113499; doi:10.1038/s41419-021-03746-0)

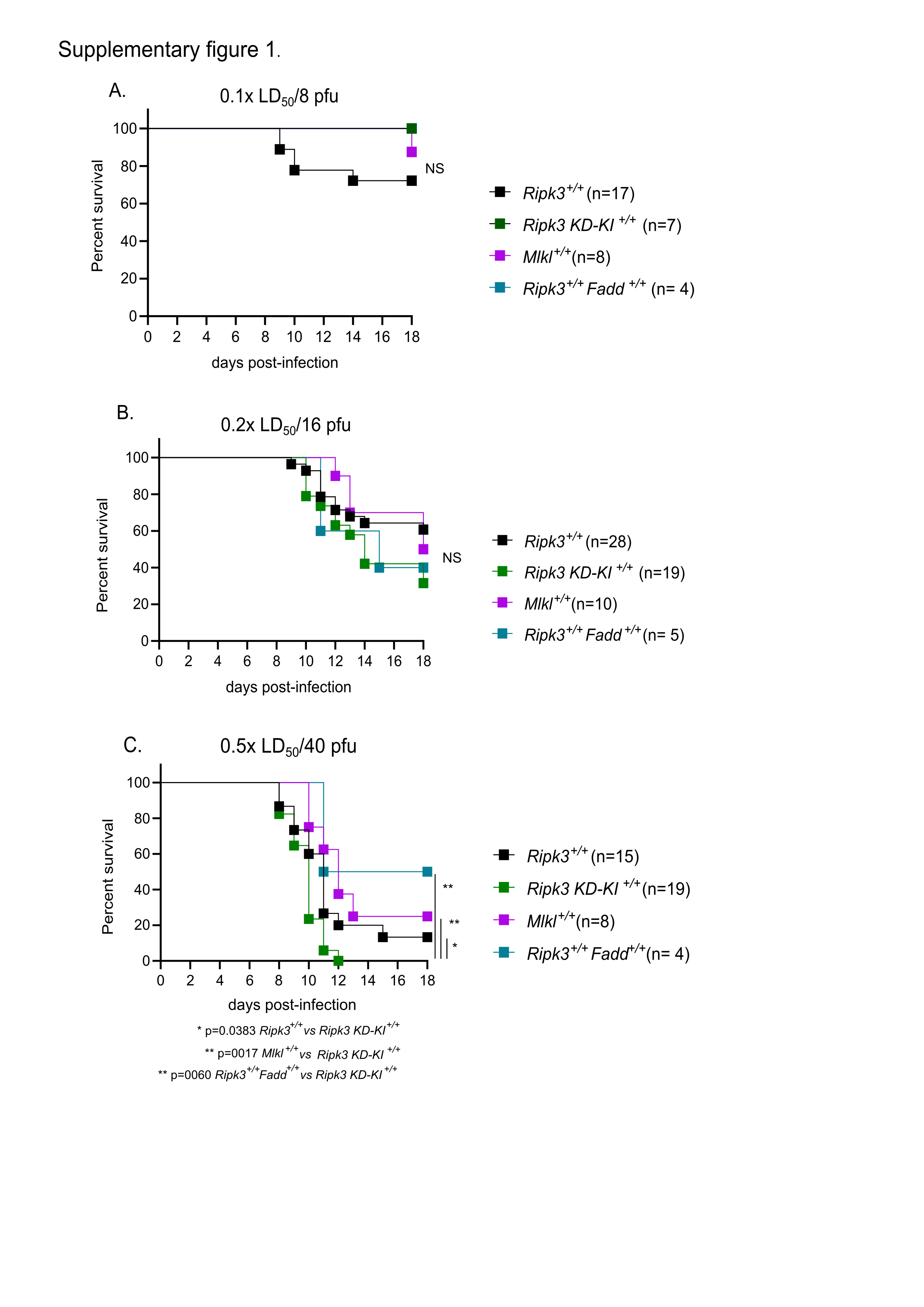

Supplement: Supplementary file 1 — Supplementary figure 1 [file 41419_2021_3746_MOESM1_ESM.png]
