# Supplementary material for: Viral dosing of influenza A infection reveals involvement of RIPK3 and FADD, but not MLKL
Source: Cell Death Dis. 2021 May 11;12(5):471. doi: 10.1038/s41419-021-03746-0 (PMC8113499; doi:10.1038/s41419-021-03746-0)
Supplement: Supplementary file 2 — Legend for supplementary figure 1 [file 41419_2021_3746_MOESM2_ESM.docx]

**Supplementary figure 1. Different WT littermate controls challenged with low, medium and high IAV doses *in vivo:*** Survival analysis of the different WT littermate controls used in this study: *Ripk3^+/+^ Mlkl^+/+^*, *Ripk3 KD-KI^+/+,^* and *Ripk3^+/+^ Fadd^+/+^*. Al mice were infected intranasally with IAV are shown: low dose: 0.1x LD_50_/8 pfu (**A**), medium dose: 0.2x LD_50_/16 pfu (**B**) and high dose: 0.5x LD_50_/40 pfu (**C**) **t**ogether with their corresponding KO mice as shown in Figure 1A-4C. Survival curves were plotted for indicated groups and evaluated statistically according to Kaplan–Meier (GraphPad Prism 7), *p < 0.05.
